# Supplementary material for: Diet-induced adaptive thermogenesis requires neuropeptide FF receptor-2 signalling
Source: Nat Commun. 2018 Nov 9;9:4722. doi: 10.1038/s41467-018-06462-0 (PMC6226433; doi:10.1038/s41467-018-06462-0)
Supplement: Supplementary file 1 — Supplementary Information [file 41467_2018_6462_MOESM1_ESM.pdf]

Supplementary information

Diet-induced adaptive thermogenesis requires Neuropeptide FF receptor-2 signalling

Zhang et al.

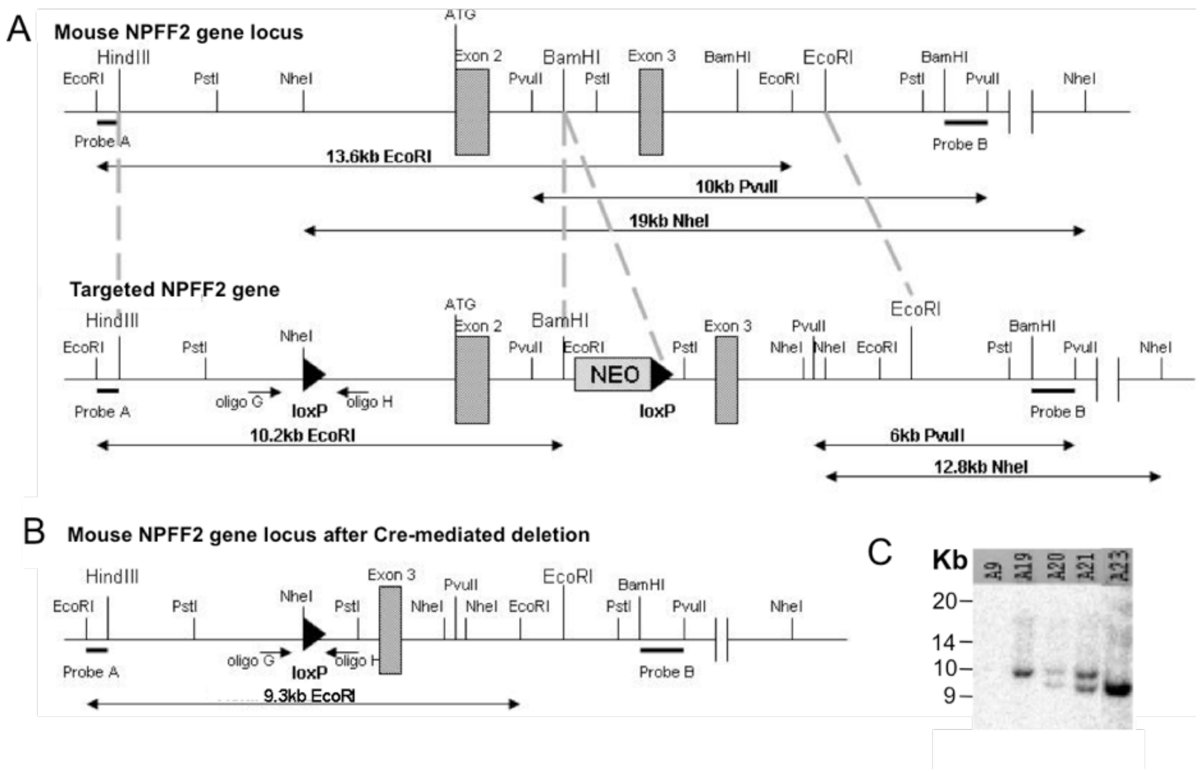

Supplemental Figure 1: Targeting strategy for NPFFR2 conditional knockout

(A) Mouse *Npffr2* gene locus and targeting construct.

(B) Re-arranged *Npffr2* locus after cre-mediated deletion.

(C) Southern Blot analysis of genomic DNA from homozygote floxed (A19), heterozygote floxed (A20, A21) and germline knockout (A23) using an EcoRI digest and hybridised with Probe-A.

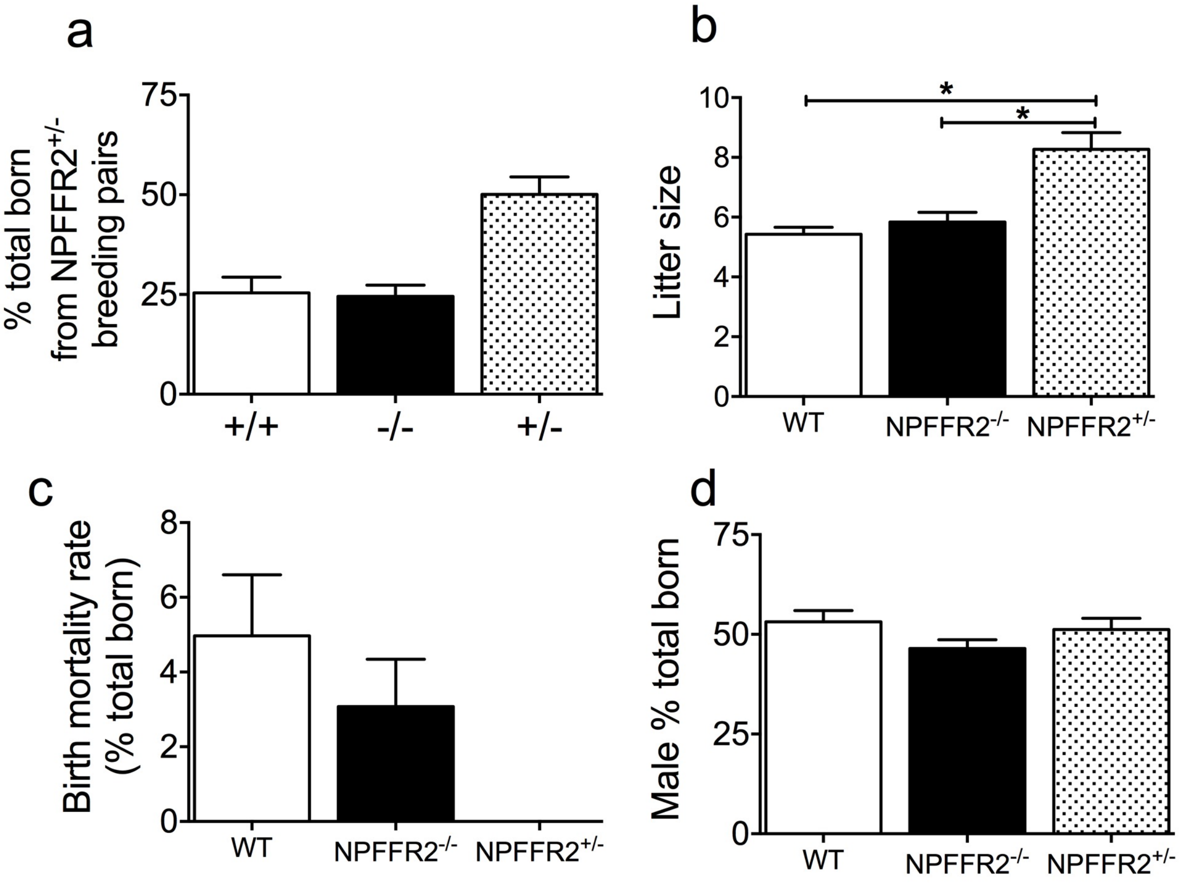

Supplementary Figure 2. Breeding profiles from WT, *Npffr2*$^{-/-}$, and *Npffr2*$^{+/-}$ breeding pairs.
(**a**) Offspring genotype composition from *Npffr2*$^{+/-}$ breeding pairs. Eight breeding pairs producing a total of 23 litters. (**b – d**) Litter size, mortality rate and gender ratio from WT, *Npffr2*$^{-/-}$, and *Npffr2*$^{+/-}$ breeding pairs. n = 8 - 10 per breeding pair. Data are mean ± SEM. One-way ANOVA was used to determine differences among groups. * $p < 0.05$ as indicated by bars.

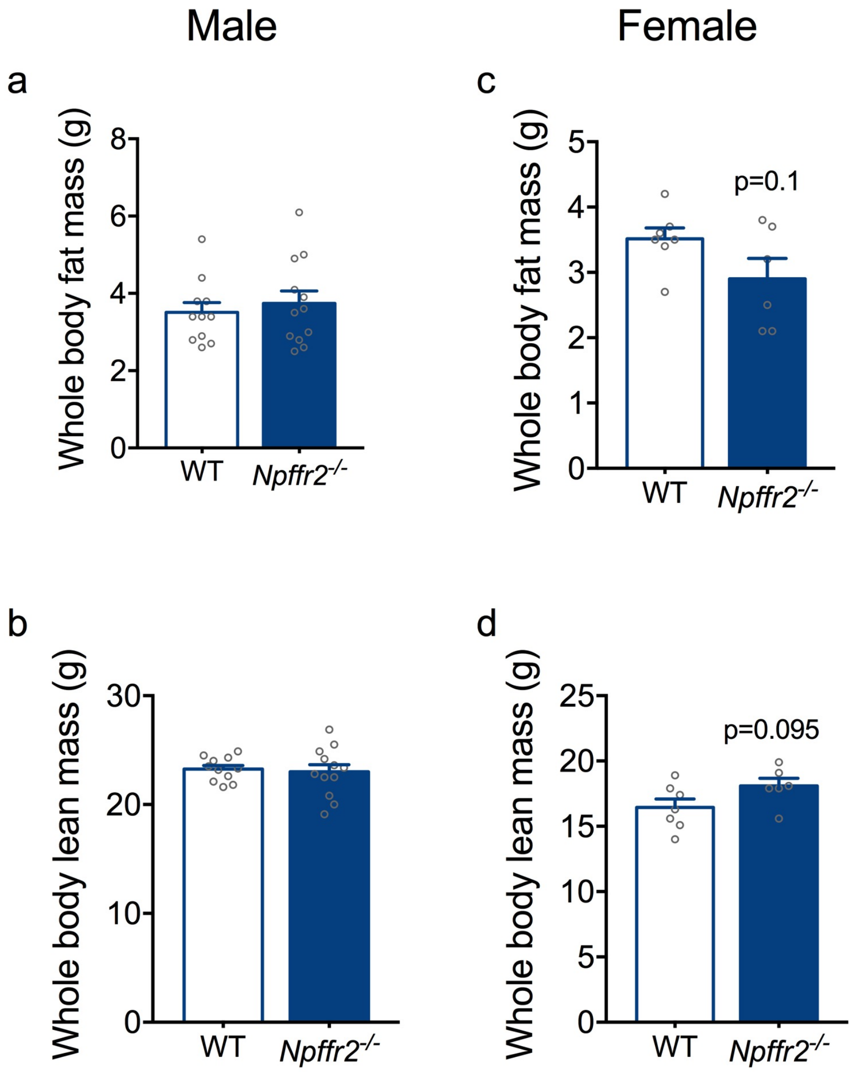

Supplementary Figure 3. Body composition assessed by dual-energy X-ray absorptiometry in chow-fed WT and *Npffr2*$^{-/-}$ mice. (**a, c**) Whole body fat mass by Dual-energy X-ray absorptiometry. (**b, d**) Whole body lean mass by Dual-energy X-ray absorptiometry. Data are mean ± SEM. Male: n = 11 and 12 for WT and *Npffr2*$^{-/-}$, respectively. Female: n = 7 and 6 for WT and *Npffr2*$^{-/-}$, respectively. One-way ANOVA was used to determine difference among groups.

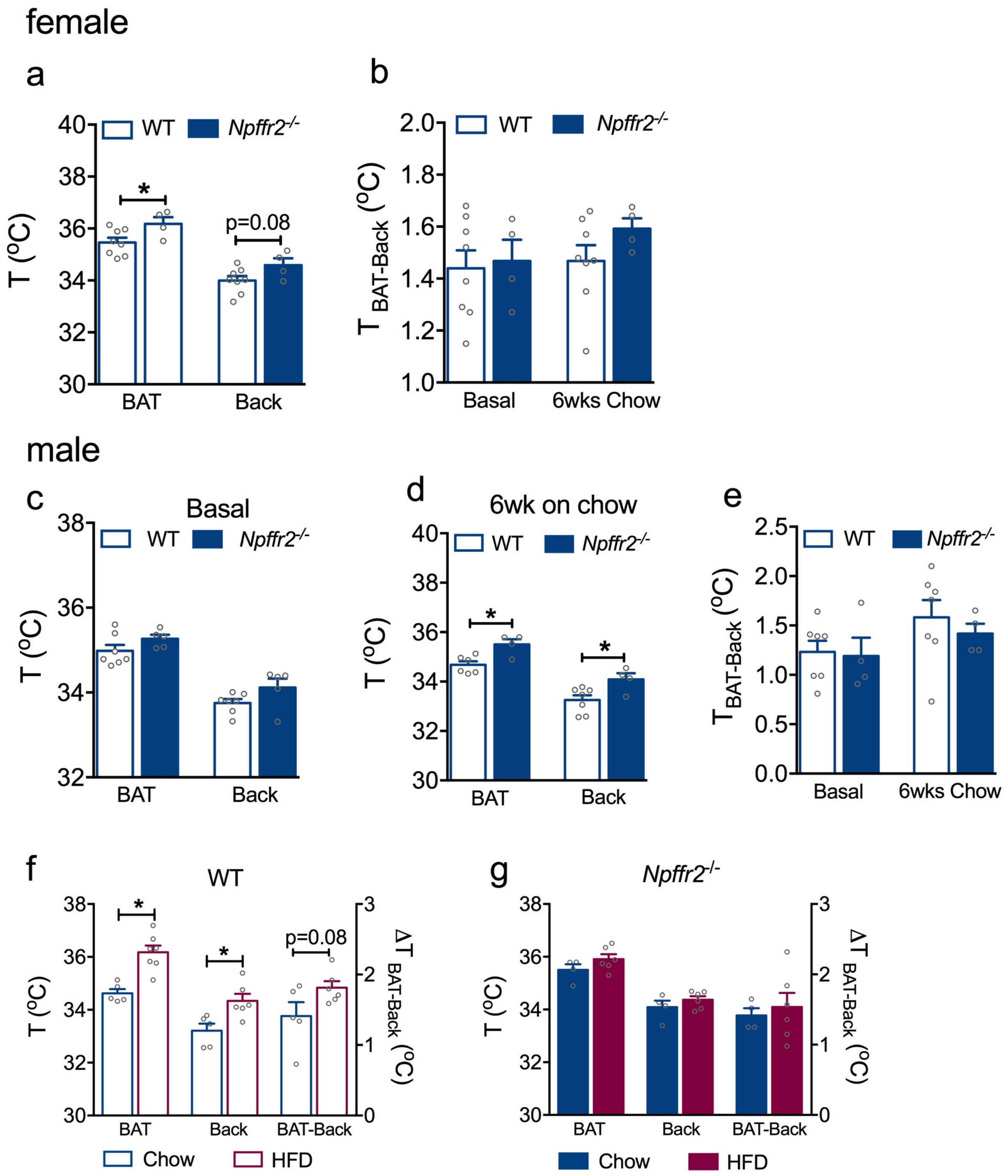

Supplementary Figure 4. Brown adipose tissue thermogenesis measured by infrared imaging. (**a, b**) T_{BAT} and T_{Back} after 6 weeks of monitoring period on chow (a) and $\Delta T_{BAT\text{-}Back}$ at baseline and after 6 weeks on chow (b) in female WT and *Npffr2*$^{-/-}$ mice. WT n = 7, *Npffr2*$^{-/-}$ n = 4. (**c, d, e**) Comparison of genotype effect on T_{BAT}, T_{Back} and $\Delta T_{BAT\text{-}Back}$ at baseline and after 6 weeks of monitoring on chow in male mice. WT n = 7, *Npffr2*$^{-/-}$ n = 4. **(f, g)** Comparison of diet effect on T_{BAT}, T_{Back} and $\Delta T_{BAT\text{-}Back}$ after 6 weeks of chow or HFD in male WT and *Npffr2*$^{-/-}$ mice. Chow: WT n = 5, *Npffr2*$^{-/-}$ n = 4; HFD: WT n = 7, *Npffr2*$^{-/-}$ n = 6. Baseline measurements were collected from mice at 14 weeks of age. Mice were then fed either chow or a HFD and monitored for 6 weeks after which the second thermal data were collected. Data are mean ± s.e.m.. One-way ANOVA was used to determine diet or genotype effects among groups. * $p < 0.05$ as indicated by bar.

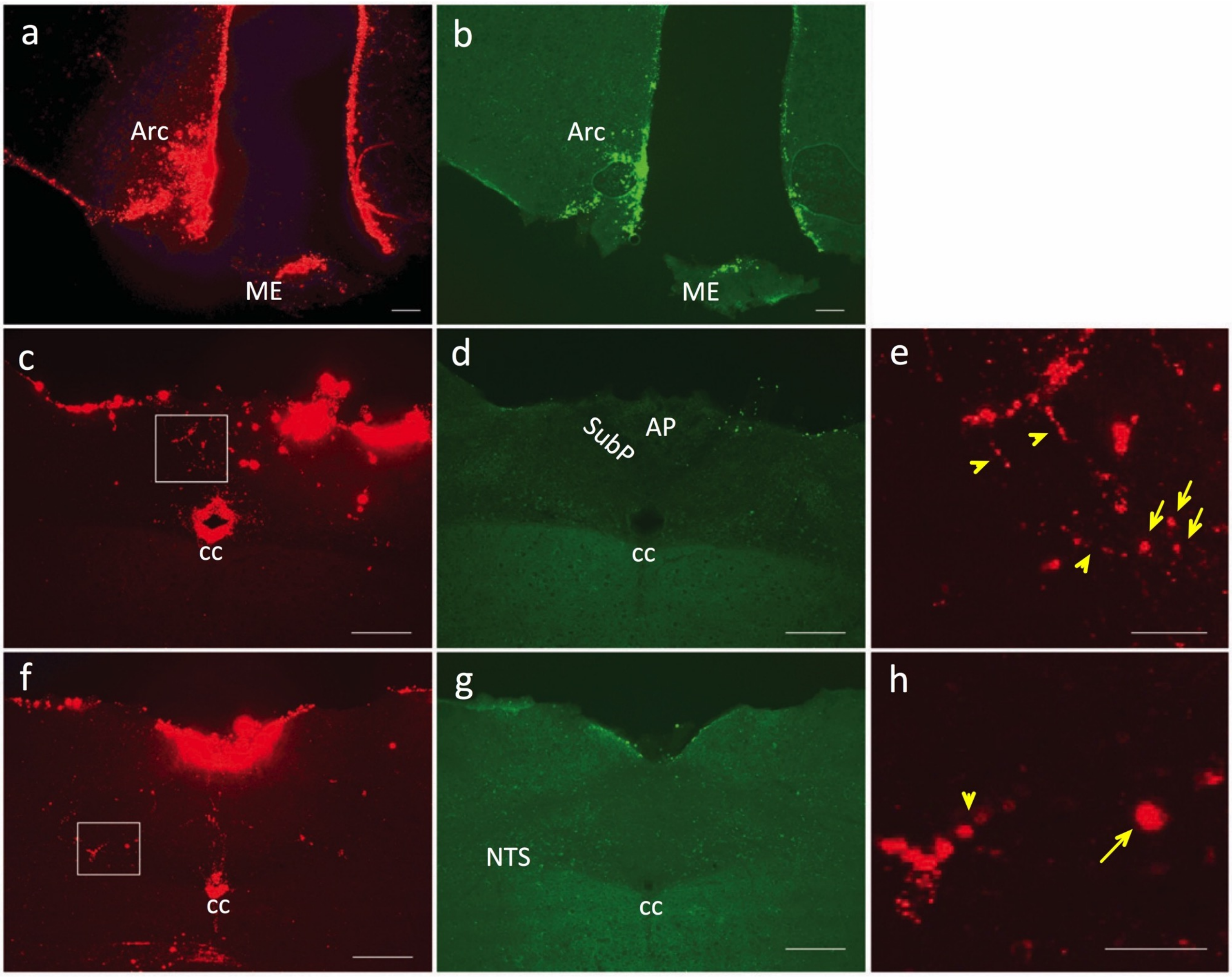

Supplementary Figure 5. **Brain stem labelling by red retro-beads after unilateral injection into mouse arcuate nucleus.** (**a,b**) Labelling at the injection site examined under red filter (a) and green filter (b). bar = 100 µm. **(c,d)** Labelling at the area postrema (AP) and subpostrema area (SubP) viewed with red filter set (c) and green filter set (d). Bar = 100 µm. (**e**) Enlarged view for window in (c). Arrow indicates labelled neuron. Arrow heads indicate labelled fibres. Bar = 25 µm. (**f,g**) Labelling at the nucleus tractus solitarius (NTS) viewed with the red (f) and the green (g) filter sets. (**h**) Enlarged view of window in (f). Arrow indicates labelled neuron. Arrow heads indicate labelled fibres. Bar = 25 µm. Representative images from 3 independents experiments. Arc: arcuate nucleus; ME : median eminence; cc: central canal.

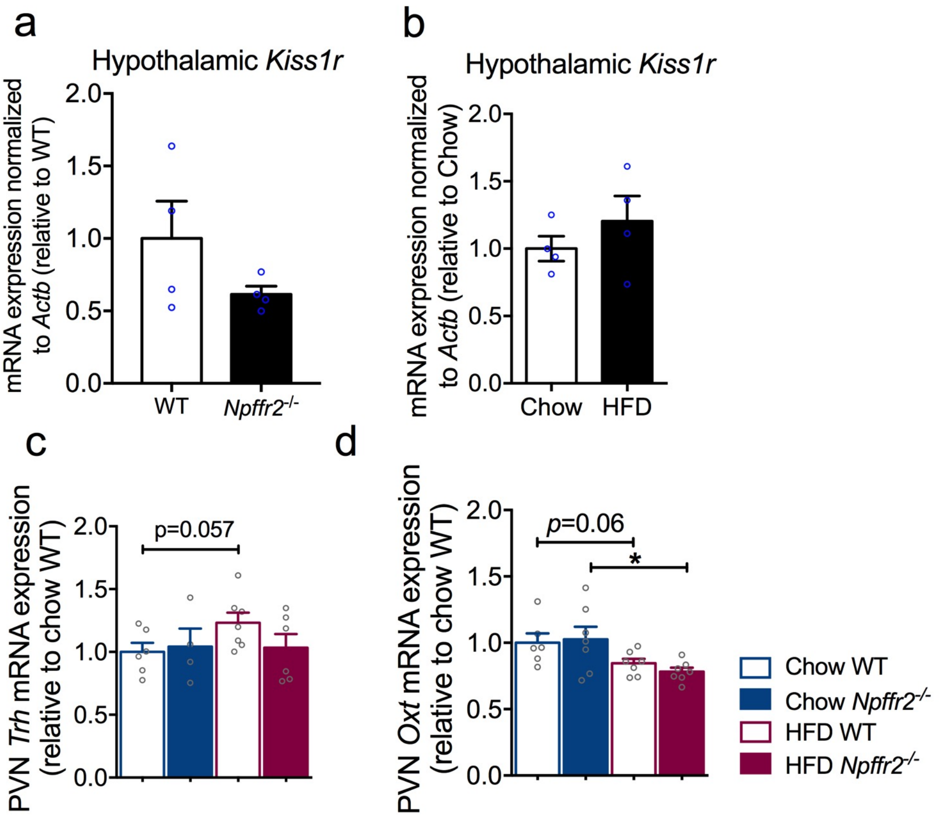

Supplementary Figure 6. qPCR analysis of *Kiss1r* mRNA expression in the hypothalamus, and ISH analysis of candidate peptides involved in energy homeostatic control. (**a, b**) Hypothalamic *Kiss1r* expression in WT and *Npffr2*$^{-/-}$ mice on chow and 9 weeks of HFD, respectively. N = 4 per group (**c, d**) Expression of thyrotropin-releasing hormone (*Trh*) and Oxytocin (*Oxt*) in the paraventricular nucleus of the hypothalamus (PVN) of WT and *Npffr2*$^{-/-}$ mice fed chow or 9 weeks of HFD diet, respectively. *Trh*: Chow (WT n=6, *Npffr2*$^{-/-}$ n=4), HFD (WT n=7, *Npffr2*$^{-/-}$ n=6). *Oxt*: Chow (WT n=6, *Npffr2*$^{-/-}$n=7), HFD (WT n=7, *Npffr2*$^{-/-}$ n=7). Data are mean ± s.e.m.. One-way ANOVA was used to determine diet or genotype effects among groups. * $p < 0.05$ as indicated by bar.

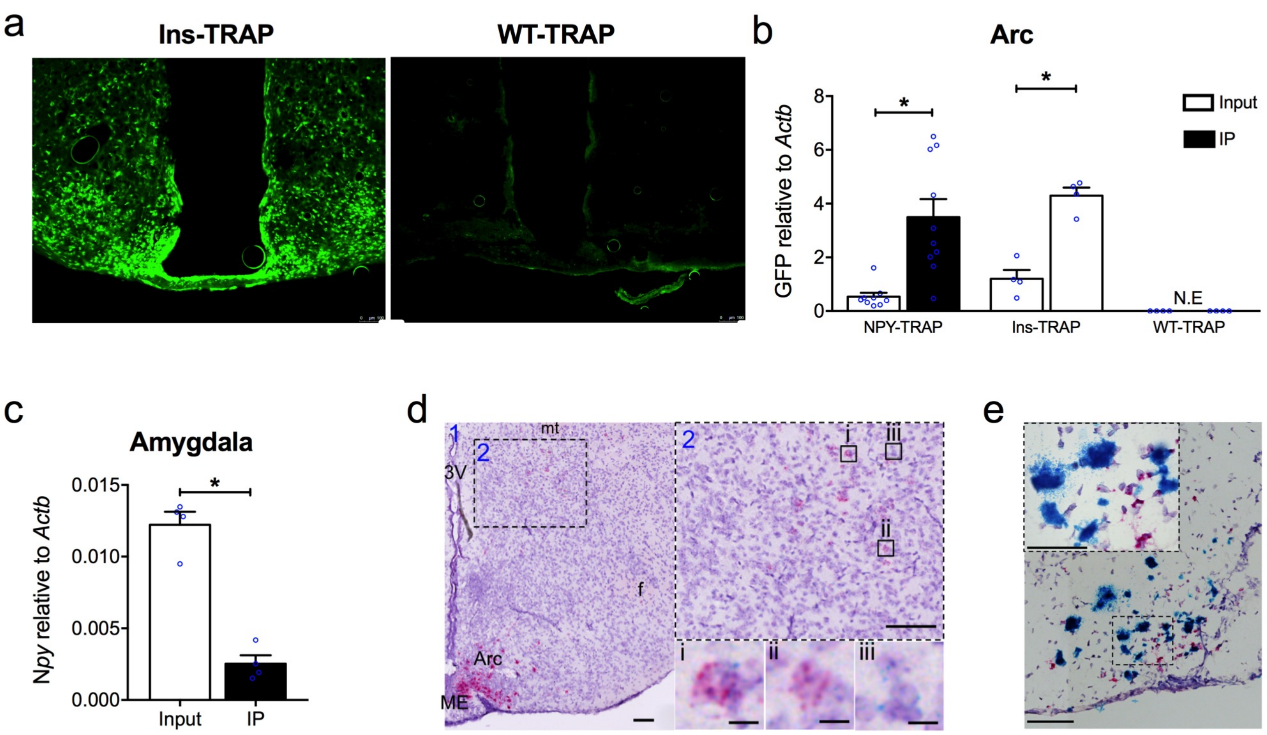

Supplementary Figure 7. Validation of TRAP and double ISH of *Npffr2* with *Npy* in dorsal medial hypothalamus or with *Pomc* in the arcuate nucleus. (**a**) Representative image of GFP expression at the arcuate nucleus of Ins-TRAP and WT-TRAP mice. Scale bar, 100µm. (**b**) Quantification of the expression of GFP mRNA in the input and immunoprecipitated (IP) RNA isolated from the arcuate nucleus of NPY-TRAP (n = 10), Ins-TRAP (n = 4) and WT-TRAP (n = 4) mice. (**c**) Quantification of the expression of *Npffr2* mRNA in the input and immunoprecipitated (IP) RNA fraction isolated from the amygdala (n = 4), N.E, gene is not expressed. One-way ANOVA was used to determine difference between groups. * $p < 0.05$ as indicated by bar. (**d)** Double chromogenic ISH assay (RNAscope) for *Npy* (red) and *Npffr2* (blue) expression in the dorsal medial hypothalamus (dashed line framed, image #2), where co-localization of *Npy* and *Npffr2* (i), as well as non-colocalizing neurons (ii and iii for *Npy*-expressing and *Npff2*-expressing neurons, respectively) were observed. Scale bar = 100 µm in images 1 and 2, 10 µm in images i), ii) and iii). (**e)** Double chromogenic ISH assay for *Pomc* (blue) and *Npffr2* (red) expression in the arcuate nucleus. Scale bar = 100 µm in main picture, bar = 50 µm in insert. Representative images from 3 independent experiments showing lack of colocalisation. mt: mammillothalamic tract; f: fornix; Arc: arcuate nucleus of the hypothalamus; ME: median eminence.

a

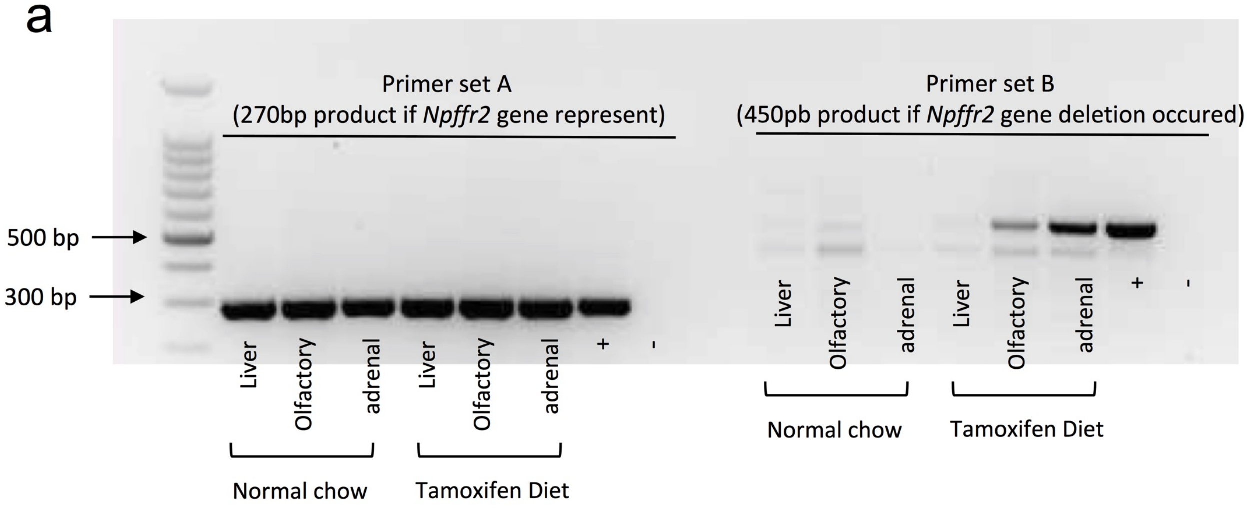

b

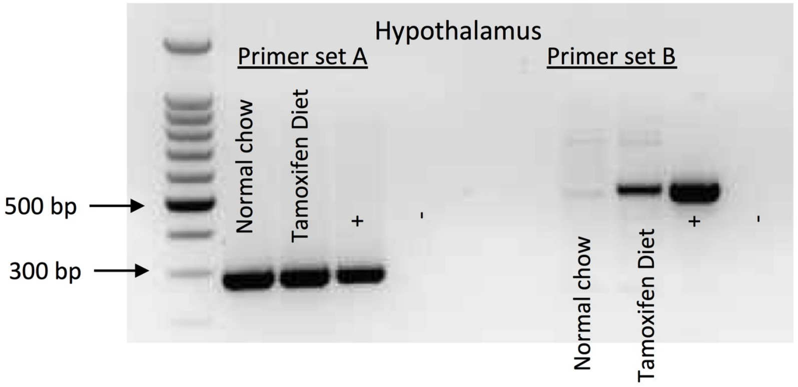

Supplementary Figure 8. Specific deletion of *Npffr2* from NPY neurons. (**a, b**) Tamoxifen-activated Cre-mediated *Npffr2* deletion in *Npy*-expressing cells was assessed by PCR from genomic DNA isolated from various peripheral tissues and the hypothalamus of conditional NPFFR2 knockout mice (*Npy*$^{creER2/+}$,*Npffr2*$^{lox/lox}$ + tamoxifen) and control mice (*Npy*$^{creER2/+}$,*Npffr2*$^{lox/lox}$). Primer set A produces a 270bp PCR product if the *Npffr2* gene is present, which was seen in all tested tissues of *Npy*$^{creER2/+}$,*Npffr2*$^{lox/lox}$ mice regardless of tamoxifen treatment status. Primer set B produces a 450bp PCR product after the *Npffr2* gene deletion, which was only seen in *Npy*-expressing tissues (i.e. olfactory, adrenal gland and hypothalamus) of tamoxifen-treated *Npy*$^{creER2/+}$,*Npffr2*$^{lox/lox}$ mice. Primer Set A: 5'-GAGCTGACCAGCAATAGCTG-3'; 5'-GGACAATCTCCAGCTACCTC-3'. Primer set B: 5'-GAGCTGACCAGCAATAGCTG-3'; 5'-CAGAGACAAGATCTCACCATG-3'.

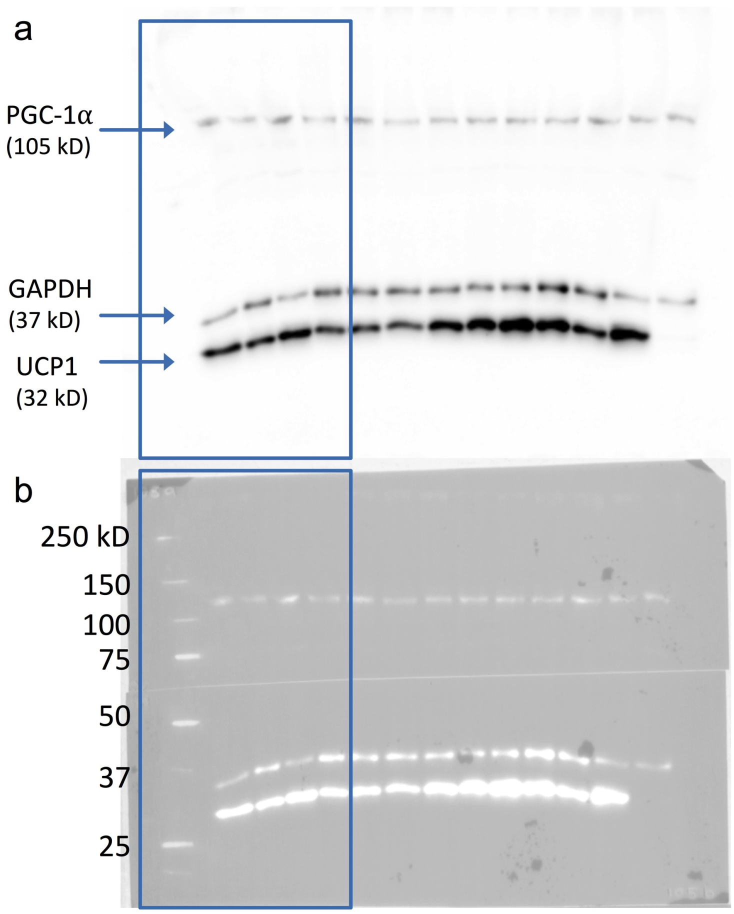

Supplementary Figure 9. Uncropped Western gel image associated with Figure 4. (**a**) Uncropped gel image of Figure 4i. Bands represented in Figure 4i are in blue frame. (**b**) Uncropped gel shown in (a) overlayed with molecular size marker.

Supplementary Table 1 Behavioural tests in male and female WT and *Npffr2*$^{-/-}$ mice. Related to Results "Generation and behavioural examination of *Npffr2*$^{-/-}$ mice".

	**Males**		**Females**	
	**WT**	**NPFFR2**$^{-/-}$	**WT**	**NPFFR2**$^{-/-}$
**Elevated Plus Maze**				
Total arm entries [n]	20.1 ± 1.9	16.4 ± 1.3	28.6 ± 1.1	25.5 ± 3.3
Open arm entries [%]	27.4 ± 3.9	39.6 ± 4.3	31.9 ± 5.1	27.1 ± 3.9
Time spent in open arms [%]	10.4 ± 2.6	19.5 ± 4.8	9.2 ± 1.6	8.2 ± 2.9
**Open Field**				
Total distance travelled [cm]	3318.6 ± 342.7	3354.4 ± 379.5	3765.8 ± 885.8	3513.3 ± 364.2
Frequency of *rearing* [n]	188.4 ± 35.5	128.6 ± 37.1	94.4 ± 38.9	75.7 ± 15.2
**Prepulse inhibition**				
ASR to 120 dB startle stimulus	53.1 ± 7.3	66.1 ± 8.7	29.7 ± 11.2	44.5 ± 6.8
%PPI for 74 dB prepulse [%]	3.5 ± 6.7	9.4 ± 7.0	-3.8 ± 8.4	12.8 ± 4.3
%PPI for 82 dB prepulse [%]	29.0 ± 4.2	36.0 ± 6.1	24.2 ± 2.9	30.7 ± 3.9
%PPI for 86 dB prepulse [%]	41.1 ± 5.3	45.0 ± 6.6	38.2 ± 4.4	43.8 ± 3.5
**Hot Water Tail Flick**				
Latency to flick the tail [s]	2.7 ± 0.2	2.6 ± 0.2	2.2 ± 0.8	1.8 ± 0.3

Data are mean ± s.e.m. of 6-8 mice per group. One-way ANOVA was used to determine difference between genotypes.

Supplementary Table 2, related to Figure. 7. Counts of NPY neurons and pCREB-ir positive neurons in the arcurate nucleus (Arc) of NPYGFP mice treated with either forskolin or a mixture of forskolin and NPFF i.c.v..

Counts per side of Arc	Forskolin (n = 5)	Forskolin+NPFF (n = 4)
NPY	95 ± 5	102 ± 6
pCREB-ir positive	272 ± 24	222 ± 15
Colocalized	58 ± 3	43 ± 3*

Brains were collected 30 minutes after the injection. Three sections between Bregma -1.7 mm and -1.94 mm were chosen from each brain and counted for the number of NPY neurons, pCREB-ir positive neurons and NPY neurons that colocalize with pCREB in the arcuate nucleus. The two sides of the Arc were counted separately, and counts (per side of Arc) from the same brain were averaged and presented. n = 5 and 4 for forskolin and forskolin+NPFF group, respectively. Data are mean ± SEM. * $p < 0.05$ vs forskolin group determined by one-way ANOVA.

Supplementary Table 3. Primer sequences

Targeted mouse gene	Sequence	System
Npvf	Mm00452052_m1	Taqman
Npff	Mm00450676_g1	Taqman
Prlh	Mm01286067_m1	Taqman
Actb	Mm00607939_s1	Taqman
Npffr2	5’- AAGCAGCATGTGCAAGATCA<br>5’- TGACAGTGAGCTTTGGCTTA	SYBR green
Kiss1r	5’- TGCTGGCTCTATATCTGCTG<br>5’- CTTGAAGCACCAGGAACAGC	SYBR green
Npy	5'-AATCAGTGTCTCAGGGCTG<br>5'-CTATCTCTGCTCGTGTGTTT	SYBR green
GFP	5'-CGGATCTTGAAGTTCACCTT<br>5'-GAGCGCACCATCTTCTTCA	SYBR green
Actb	5'-GTACGACCAGAGGCATACA<br>5'-AGCACCCTGTGCTGCTCA	SYBR green
Npy	5’-<br>GAGGGTCAGTCCACACAGCCCCATTCGC<br>TTGTTACCTAGCAT-3’	Radiolabeled *in situ* hybrydization
Pomc	5’-<br>TGGCTGCTCTCCAGGCACCAGCTCCACAC<br>ATCTATGGAGG-3’	Radiolabeled *in situ* hybrydization
Trh	5’-<br>AACCTTACTCCTCCAGAGGTTCCCTGACC<br>CAGGCTTCCAGTTGTG-3’	Radiolabeled *in situ* hybrydization
Th	5’-<br>CTCTAAGGAGCGCCGGATGGTGTGAGGA<br>CTGTCCAGTACATCA-3’	Radiolabeled *in situ* hybrydization
Npff	5’-<br>GAGACTGAGGAAGGCACAGGCAAGCAA<br>GGAGCCATGAACCACAGG-3’	Radiolabeled *in situ* hybrydization
Prlh	5’-<br>GCTGTGAGAGAACTTGGCACTTCCATCCA<br>GTGGGAAGCAGCTTAG-3’	Radiolabeled *in situ* hybrydization
Npy	ACD #313321-C2	RNAscope®
Npffr2	ACD #410171	RNAscope®
Pomc	ACD #314081-C2	RNAscope®
Lepr	ACD #402731	RNAscope®
Npff	ACD #300031-C2	RNAscope®
